# Attitudes toward early palliative care in cancer patients and caregivers: a Korean nationwide survey

**DOI:** 10.1002/cam4.1441

**Published:** 2018-03-25

**Authors:** Shin Hye Yoo, Miso Kim, Young Ho Yun, Bhumsuk Keam, Young Ae Kim, Yu Jung Kim, Hyun‐Jeong Shim, Eun‐Kee Song, Jung Hun Kang, Jung Hye Kwon, Jung Lim Lee, Soon Nam Lee, Si‐Young Kim, Eun Joo Kang, Young Rok Do, Yoon Seok Choi, Kyung Hae Jung

**Affiliations:** ^1^ Department of Internal Medicine Seoul National University Hospital Seoul Korea; ^2^ Department of Family Medicine Seoul National University College of Medicine Seoul Korea; ^3^ Cancer Survivorship Branch National Cancer Control Institute National Cancer Center Goyang Korea; ^4^ Department of Internal Medicine Seoul National University Bundang Hospital Seoul National University College of Medicine Seongnam Korea; ^5^ Division of Hematology and Medical Oncology Department of Internal Medicine Chonnam National University School of Medicine Hwasun Korea; ^6^ Division of Hematology/Oncology Chonbuk National University Medical School Jeonju Korea; ^7^ Department of Internal Medicine Postgraduate Medical School Gyeongsang National University Jinju Korea; ^8^ Department of Internal Medicine Kangdong Sacred Heart Hospital Hallym University Seoul Korea; ^9^ Department of Hemato‐oncology Daegu Fatima Hospital Daegu Korea; ^10^ Department of Internal Medicine Ewha Womans University College of Medicine Seoul Korea; ^11^ Departments of Medical Oncology and Hematology Kyung Hee University Hospital Seoul Korea; ^12^ Department of Internal Medicine Korea University Guro Hospital Seoul Korea; ^13^ Departments of Internal Medicine Dongsan Medical Center Keimyung University School of Medicine Daegu Korea; ^14^ Departments of Internal Medicine Chungnam National University Hospital Daejeon Korea; ^15^ Departments of Internal Medicine Asan Medical Center Ulsan University College of Medicine Seoul Korea

**Keywords:** Attitude, cancer, caregiver, early palliative care, patient

## Abstract

Integrated early palliative care (EPC) improves quality of life and reduces psychological distress in adult patients with cancer and caregivers, but attitudes toward EPC have been poorly studied. We aimed to investigate attitudes toward EPC in a nationwide survey of patients with cancer and caregivers. From July to October 2016, we administered nationwide questionnaires examining attitudes toward EPC in patients with cancer (*n* = 1001) and their families (*n* = 1006) from 12 Korean hospitals. When an individual considered EPC unnecessary, the reasons were collected and analyzed. Factors associated with perception of EPC were examined. A majority of patients (84.5%) and caregivers (89.5%) had positive attitudes toward EPC. The most common reasons for deeming EPC unnecessary were that EPC may be an obstacle to cancer treatment (patients: 37%; caregivers: 23%; respectively) or that they were not sure if EPC is beneficial (patients: 21%; caregivers: 24%; respectively). Financial burden as a reason was more evident in caregivers (23%) than in patients (17%). Male gender, age <50, early stage, intensive care unit admission, and not believing that dying people should prepare to practice charity were associated with patients’ negative attitudes. In caregivers, opposition to EPC was associated with not thinking death should be feared, not thinking people should be remembered, and lower educational level. Our findings showed that significant numbers of patients with advanced cancer and family caregivers showed positive attitudes toward EPC. However, more than 10% of participants did not consider EPC necessary. Physicians’ communication with patients and caregivers and financial support could help overcome the barriers of EPC.

## Introduction

Patients with advanced cancer struggle with huge physical, psychological, socioeconomical, and mental health burdens. Needs of patients and caregivers for palliative care (PC) to alleviate these problems are growing, but PC is usually provided only as end‐of‐life hospice care 1, 2, causing significant unmet need in patients with advanced cancer.

Early integration of PC into oncologic care, which is called early palliative care (EPC), indicates that PC should be administered to patients with advanced cancer earlier than in current practice, such as within 8 weeks after cancer diagnosis 3, 4, 5, 6. According to recent reports, EPC improves quality of life, psychological distresses, and understanding of disease and treatment compared to standard oncologic care alone 7, 8, 9. Moreover, one study demonstrated a survival gain associated with EPC 7. Based on these studies, several reviews and guidelines, such as the recent update to the *American Society of Clinical Oncology Clinical Practice Guidelines* 4, 5, 6, recommend that PC should be concurrently provided with standard oncologic care in patients with advanced non‐small‐cell lung cancer.

However, EPC has not been routinely integrated into cancer management as a part of standard practice in Asian countries, including Korea 10. To promote this integration, it is important to understand the attitudes and perceptions of patients with cancer and family caregivers. Although attending physicians can influence early integration by determining the timing of PC referral, understanding patient and caregiver perceptions of EPC may help reduce the physician factors that are a barrier to EPC. Some physicians may avoid early referral to PC services due to a belief that patients and caregivers may react with concern about not receiving active anticancer treatments 11, 12. However, there has been little investigation into the attitudes toward EPC in adult patients with cancer and family, although a survey of pediatric patients with cancer and parents reported that few opposed receiving EPC 13. If the patient and caregiver reaction to EPC is optimistic, then physicians may advocate for PC integration at earlier stages of disease. Hence, we aimed to examine the attitudes toward EPC in patients with cancer and family caregivers in a nationwide study.

## Methods

### Participants and procedures

Patients with cancer and family caregivers were surveyed from 12 large hospitals in South Korea from July to October in 2016. Medical oncologists were asked to identify patients with cancer at outpatient clinics. The process of participant selection was previously described in detail 14. Patients and caregivers who consented to participate were eligible if they were ≥18 years old, could fill out the questionnaire by themselves and communicate well with assistants, and understood the purpose of this study. Of 6024 consecutive patients surveyed, 1001 patients with cancer were included in our study (response rate 16.6%). For each patient at the outpatient clinic, a research assistant provided information about the study to the patient's primary caregiver. Of 5017 total family caregivers, we included 1006 in our study (response rate 20.1%). We did not include patients and caregivers from the community in this study. As part of the informed consent process, a trained research assistant provided a document written in standard Korean language that elaborated the study's objectives. The participant was then asked to comment on their understanding of the document. If the participant had speech or hearing limitations, he or she was then excluded from the study. Other than this screening, we did not test the cognitive capacity of participants in the questionnaire. Patients and caregivers who were included received information about the study and completed a self‐reporting questionnaire with the aid of research assistants. All provided written informed consent, and the study was approved by the institutional review board of each hospital (IRB Number: E‐1612‐102‐815). We conducted the study in accordance with the principles of the Declaration of Helsinki.

### Measurements

The structured questionnaire utilized in the survey included this question: “Currently, palliative care service is only provided for patients in a terminal state in Korea. Some people insist that palliative care should be provided earlier than the terminal stages of illness. Early palliative care is defined as palliative care services that try to control symptoms, such as pain, and to give emotional and psychosocial support before the terminal state, even as early as diagnosis. Do you think early palliative care is necessary?” Scores ranged from 1 to 4 (1 = strongly necessary, 2 = necessary, 3 = unnecessary, and 4 = strongly unnecessary). We also examined reasons for considering EPC unnecessary. The following seven possible answers were provided: (1) I am satisfied with current treatment, (2) early palliative care may be an obstacle to cancer treatment, (3) early palliative care is an additional burden of time and effort, (4) early palliative care is an additional financial burden, (5) I do not know about palliative care, (6) I am not sure if palliative care is beneficial, and (7) my attending physician does not recommend early palliative care. If the respondents wanted to provide a different reason than one listed, they could write a new answer in a blank eighth space. The term “terminal state” was defined on the questionnaire as a state of progressive advanced disease that, in the physician's judgment, was refractory to conventional anticancer treatments, such as surgery, chemotherapy, radiotherapy, or hormone therapy, and wherein the patient was expected to die within months. We have modified the definitions of PC by the World Health Organization 15 and the National Comprehensive Cancer Network 16 and specified the following definitions in the questionnaire: “Palliative care is an approach that focuses on the management of pain and other distressing symptoms and provides patients and their families with comprehensive psychosocial and spiritual care. In addition, palliative care experts, including social workers and chaplains, work together as an interdisciplinary team.” Five domains of self‐rated health status—physical, mental, social, spiritual, and general—were each evaluated on a scale of 1–5 (Excellent, Very Good, Good, Poor, and Very Poor). Details of attitudes toward dying and death were described in a previous report 14. Willingness to communicate with family about dying and death was scored from 1 to 4 (Very Much, Much, Little, and Never). Sociodemographic information (age, sex, level of education and income, current job status, religion, type of insurance, and status of private insurance) was examined for both patients and caregivers, whereas clinical information (site of malignancy, stage, treatment status, Eastern Cooperative Oncology Group [ECOG] performance status, experience of emergency room visit, and intensive care unit [ICU] admission) was investigated only for patients.

### Statistical analysis

Sociodemographic and clinical characteristics of patients and caregivers were presented as numbers and percentages. Attitudes toward EPC were compared using chi‐square tests between two groups. The response to any question as “strongly necessary” or “necessary” was considered as having a positive attitude in that category. We treated answers to questions about attitudes of early palliative care as dichotomous outcomes: either positive or negative. Self‐rated health status was dichotomized (More than Good [Excellent, Very Good, or Good] vs. Not Good [Poor or Very Poor]). We investigated the associations of sociodemographic and clinical characteristics, self‐rated health status, and attitudes toward dying and death with negative attitudes toward EPC separately for patients with cancer and caregivers. Because most factors included in the analysis were categorical variables, a normality test was unnecessary. We also did not need to test for normality because we transformed continuous variables to categorical variables to perform simple comparisons and logistic regression analyses. Univariate comparisons were performed using chi‐square tests. Backward‐selected multivariable logistic regression analysis in models that included statistically significant variables significant from the univariate analysis with *P *<* *0.10 was utilized to identify predictors for negative attitude for EPC. The number of missing values from all dependent and independent variables was very low (34 of 40,125 [0.08%]). Because we did not observe skewing in the missing values, the occurrence of missing data was assumed to be randomly distributed. Thus, we performed an available case analysis. To identify any outliers of the continuous variables, we drew a distribution of continuous variables and found no outliers. We used STATA version 12 software (StataCorp LP, College Station, TX, USA) for all analyses and calculated two‐sided *P*‐values that were considered significant when *P *<* *0.05.

## Results

### Baseline characteristics of patients with cancer and family caregivers

A total of 2007 participants were included in this analysis. Sociodemographic and clinical characteristics of patients with cancer and family caregivers are shown in Table 1.

**Table 1:** Sociodemographic and clinical characteristics of (A) 2007 people (B) 1001 patients with cancer who participated in a survey about attitudes toward early palliative care

Variables	Cancer patient *n *=* *1001	Family caregiver *n *=* *1006
(A)		
Gender		
Male	390 (39.0)	324 (32.2)
Female	610 (61.0)	682 (67.8)
Age, years		
<50	334 (33.4)	596 (59.2)
≥50	667 (66.6)	409 (40.8)
Caregiver relationship with patient		
Parents, grandparents, sibling, relatives	NA	196 (19.5)
Children, grandchildren, children‐in‐law		490 (48.7)
Spouse		320 (31.8)
Level of education		
Middle school or less	205 (20.6)	75 (7.4)
High school	433 (43.5)	401 (39.9)
College or higher	358 (35.9)	530 (52.7)
Marital status		
Single/separated/widowed/divorced	212 (21.1)	195 (19.4)
Married	789 (78.9)	811 (80.6)
Current job status		
No	737 (74.1)	569 (56.6)
Yes	257 (25.9)	437 (43.4)
Presence of religion		
No	462 (46.3)	494 (49.1)
Yes	536 (53.7)	512 (50.9)
Monthly income (in 1000 Korean won)		
<2000	260 (26.0)	117 (11.6)
2000–2999	196 (19.6)	183 (18.2)
3000–3999	217 (21.7)	260 (25.8)
≥4000	328 (32.7)	446 (44.4)
Type of insurance		
National Health Insurance	931 (93.1)	941 (93.7)
Medical aid	69 (6.9)	63 (6.3)
Presence of private insurance		
Yes	717 (71.8)	878 (87.5)
No	282 (28.2)	126 (12.5)
Comorbidity		
Yes	70 (7.0)^a^	77 (7.7)
No	931 (93.0)	929 (92.3)
Caregiver experience^b^		
Yes	233 (23.3)	990 (98.4)
No	766 (76.7)	16 (1.6)
(B)		
Cancer type		
Stomach cancer	147 (14.7)	NA
Lung cancer	104 (10.4)	
Hepato‐pancreato‐biliary cancer	101 (10.1)	
Colorectal cancer	128 (12.8)	
Breast cancer	229 (22.9)	
Hematologic malignancy	106 (10.6)	
Others^c^	184 (18.4)	
Time from diagnosis to survey		
Mean ± SD, months	26.7 ± 33.1	NA
≥5 years	121 (12.1)	NA
<5 years	878 (87.9)	
Stage		
I	148 (14.8)	NA
II	303 (30.3)	
III	325 (32.5)	
IV	157 (15.7)	
Other	68 (6.7)	
Treatment status		
Diagnosis ~ treatment (in progress)	715 (71.5)	NA
Treatment (completed) ~ remission	243 (24.3)	
Not cured or terminal	42 (4.2)	
ECOG performance status		
0	285 (28.5)	NA
1	510 (51.0)	
2–4	205 (20.5)	
Experience of ER visit due to cancer		
Yes	294 (29.4)	NA
No	705 (70.6)	
Experience of ICU admission due to cancer		
Yes	219 (21.9)	NA
No	780 (78.1)	

NA, not applicable; SD, standard deviation; ECOG, the Eastern Cooperative Oncology Group; ER, emergency room; ICU, intensive care unit.

a For patients with cancer, we counted the number of nonmalignant comorbidities including human immunodeficiency virus infection, chronic disease including diabetes mellitus, hypertension, lung disease, liver disease, heart failure, kidney disease, and arthritis (rheumatoid arthritis, osteoarthritis), stroke, Parkinson's disease, dementia, and incurable genetic and neurologic disorders.

b Caregiver experience means the presence of illness experience of loved one.

c Others include cancers of uterus, ovary, prostate, genitourinary except prostate, testis, central nervous system, head and neck, esophagus, and thyroid, germ cell tumor, osteosarcoma, skin cancer (melanoma, nonmelanoma), and neuroendocrine tumor.

### Attitudes toward early palliative care

**Figure 1:**
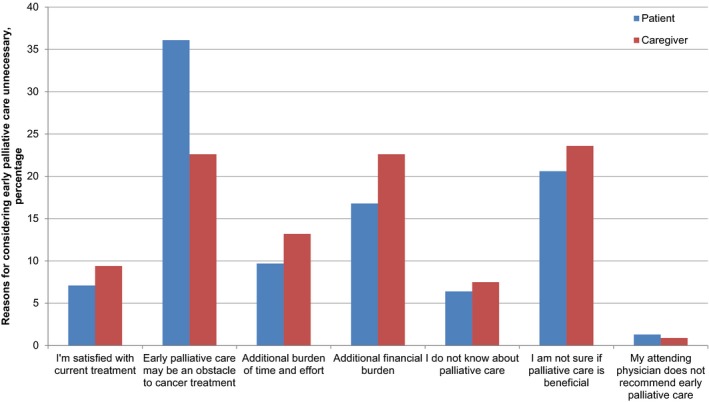
Reasons provided by patients with cancer and family caregivers for considering early palliative care to be unnecessary.

Positive attitude toward EPC was evident in both patients (84.5%, *n* = 846) and caregivers (89.5%, *n* = 900), and more likely to be seen in caregivers (*P *<* *0.001). For patients and caregivers who thought that EPC was unnecessary or strongly unnecessary, the reasons are shown in Figure 1. Patients presuming EPC unnecessary were frequently worried whether EPC may be an obstacle to cancer treatment (37%, *n* = 56) or not sure whether EPC is beneficial (21%, *n* = 32). These reasons were also commonly reported by the caregivers (23%, *n* = 24, and 24%, *n* = 25, respectively). Financial burden as a reason was noted more in caregivers (23%, *n* = 24) than in patients (17%, *n* = 26). Other reasons for negative attitudes toward EPC were additional burden of time and effort, 10% (*n* = 15) in patients and 13% (*n* = 14) in caregivers; satisfaction with current treatment, 7% (*n* = 11) in patients and 9% (*n* = 10) in caregivers; unawareness of PC, 7% (*n* = 10) in patients and 7% (*n* = 8) in caregivers; and attending physician's nonrecommendation, 1% (*n* = 2) in patients and 1% (*n* = 1) in caregivers.

### Factors associated with negative attitudes toward early palliative care

We analyzed factors associated with negative attitudes toward EPC separately for patients and caregivers by sociodemographic and clinical characteristics, self‐rated health status, and attitude toward death. For patients, negative attitude toward EPC was associated with male sex, age <50 years, employment, the absence of religion, early cancer stage, and having experienced ICU admission. For caregivers, those who were older than 50 years, had a lower educational level, and earned less showed more negative attitudes toward EPC (Table 2). Poor physical health status, mental health status, and general health status were associated with negative attitude toward EPC among patients, whereas this was true of poor mental, social, and spiritual health status among caregivers (Table 3). For patients, negative attitude toward EPC was associated with a negative response regarding whether dying people should prepare to practice charity. Among caregivers, negative attitude toward EPC was associated with disagreement with the following ideas: death as the ending of life, death is painful and therefore to be feared, and people should be remembered (Table 3).

**Table 2:** Factors associated with negative attitude toward early palliative care by sociodemographic and clinical characteristics

Factor	Variables	Patient			Caregivers		
		Positive	Negative	*P* value	Positive	Negative	*P* value
Sociodemographic variables							
Sex	Male	307 (78.7)	83 (21.3)	<0.001	288 (88.9)	36 (11.1)	0.683
	Female	538 (88.2)	72 (11.8)		612 (89.7)	70 (10.3)	
Age	<50	268 (80.2)	66 (19.8)	0.008	543 (91.1)	53 (8.9)	0.041
	≥50	578 (86.7)	89 (13.3)		357 (87.1)	53 (12.9)	
Caregiver relationship with patient	Parents, grandparents, sibling, relatives	NA			182 (92.9)	14 (7.1)	0.225
	Children, grandchildren, children‐in‐law				434 (88.6)	56 (11.4)	
	Spouse				284 (88.7)	36 (11.3)	
Education	Middle school or less	171 (83.4)	34 (16.6)	0.575	52 (69.3)	23 (30.7)	<0.001
	High school	372 (85.9)	61 (14.1)		370 (92.3)	31 (7.7)	
	College or higher	299 (83.5)	59 (16.5)		478 (90.2)	52 (9.8)	
Marital status	Single/separated/widowed/divorced	177 (83.9)	34 (16.1)	0.782	170 (87.2)	25 (12.8)	0.247
	Married	668 (84.7)	121 (15.3)		730 (90.0)	81 (10.0)	
Current job status	No	637 (86.4)	100 (13.6)	0.003	512 (90.0)	57 (10.0)	0.541
	Yes	202 (78.6)	55 (21.4)		388 (88.8)	49 (11.2)	
Religion	No	379 (82.0)	83 (18.0)	0.040	437 (88.5)	57 (11.5)	0.309
	Yes	465 (86.8)	71 (13.2)		463 (90.4)	49 (9.6)	
Monthly income (in 1000 Korean won)	<2000	221 (85.0)	39 (15.0)	0.864	96 (82.1)	21 (17.9)	0.010
	2000–3999	346 (83.8)	67 (16.2)		395 (89.2)	48 (10.8)	
	≥4000	279 (85.1)	49 (14.9)		409 (91.7)	37 (8.3)	
Type of insurance	National insurance	785 (84.3)	146 (15.7)	0.560	839 (89.2)	102 (10.8)	0.139
	Medical aid	60 (87.0)	9 (13.0)		60 (95.2)	3 (4.8)	
Presence of private insurance	Yes	601 (83.8)	117 (16.2)	0.357	784 (89.3)	94 (10.7)	0.499
	No	243 (86.3)	39 (13.8)		115 (91.3)	11 (8.7)	
Comorbidity	Yes	NA			70 (90.9)	7 (9.1)	0.668
	No				830 (89.3)	99 (10.7)	
Caregiver experience	Yes	201 (86.3)	32 (13.7)	0.391	885 (89.4)	105 (10.6)	0.579
	No	643 (83.9)	123 (16.1)		15 (93.8)	1 (6.2)	
Clinical variables (patient only)							
Time to survey from diagnosis	≥5 years	108 (89.3)	13 (10.7)	0.125	NA		
	<5 years	736 (83.8)	142 (16.2)				
Stage	Advanced	419 (86.9)	63 (13.1)	0.043	NA		
	Early	427 (82.3)	92 (17.7)				
Treatment status	Diagnosis ~ treatment (in progress)	603 (84.3)	112 (15.7)	0.233	NA		
	Treatment (completed) ~ remission	210 (86.4)	33 (13.6)				
	Not cured or terminal	32 (76.2)	10 (23.8)				
ECOG performance status	0	240 (84.2)	45 (15.8)	0.453	NA		
	1	437 (85.7)	73 (14.3)				
	2–4	168 (81.9)	37 (18.1)				
Experience of ER visit due to cancer	No	599 (85.0)	106 (15.0)	0.517	NA		
	Yes	245 (83.3)	49 (16.7)				
Experience of ICU admission due to cancer	No	669 (85.8)	111 (14.2)	0.035	NA		
	Yes	175 (79.9)	44 (20.1)				

ECOG, the Eastern Cooperative Oncology Group; ER, emergency room; ICU, intensive care unit; NA, not applicable.

*P* value was estimated by chi‐square test or Fisher's exact test, in some cases the cell value <5%.

**Table 3:** Factors associated with negative attitude toward early palliative care by self‐rated health status and attitude toward dying and death

Factor	Variables	Patient			Caregivers		
		Positive	Negative	*P* value	Positive	Negative	*P* value
Self‐rated health status^a^							
Self‐rated physical health	More than good	285 (87.4)	41 (12.6)	0.078	756 (90.0)	84 (10.0)	0.214
	Not good	561 (83.1)	114 (16.9)		144 (86.7)	22 (13.2)	
Self‐rated mental health	More than good	493 (86.5)	77 (13.5)	0.048	692 (90.6)	72 (9.4)	0.042
	Not good	353 (81.9)	78 (18.1)		208 (85.9)	34 (14.1)	
Self‐rated social health	More than good	518 (85.6)	87 (14.4)	0.233	789 (90.2)	86 (9.8)	0.061
	Not good	328 (82.8)	68 (17.2)		111 (84.7)	20 (15.3)	
Self‐rated spiritual health	More than good	546 (85.3)	94 (14.7)	0.354	768 (90.2)	83 (9.8)	0.060
	Not good	300 (83.1)	61 (16.9)		132 (85.2)	23 (14.8)	
Self‐rated general health	More than good	485 (86.6)	75 (13.4)	0.040	787 (89.9)	88 (10.1)	0.202
	Not good	361 (81.9)	80 (18.1)		113 (86.3)	18 (13.7)	
Attitude toward dying and death^b^							
Life ends with death	Positive	644 (84.4)	119 (15.6)	0.861	672 (90.4)	71 (9.6)	0.090
	Negative	202 (84.9)	36 (15.1)		228 (86.7)	35 (13.3)	
Death is painful and therefore to be feared	Positive	495 (85.6)	83 (14.4)	0.251	531 (90.9)	53 (9.1)	0.077
	Negative	351 (83.0)	82 (17.0)		369 (87.4)	53 (12.6)	
Life continues after death	Positive	465 (86.1)	75 (13.9)	0.132	496 (90.4)	53 (9.6)	0.318
	Negative	381 (82.6)	80 (17.4)		404 (88.4)	53 (11.6)	
Dying people should prepare to practice charity	Positive	774 (85.9)	127 (14.1)	<0.001	820 (89.4)	97 (10.6)	0.891
	Negative	72 (72.0)	28 (28.0)		80 (89.9)	9 (10.1)	
People should be remembered	Positive	776 (84.4)	143 (15.6)	0.824	825 (90.1)	91 (9.9)	0.050
	Negative	70 (85.4)	12 (14.6)		75 (83.3)	15 (16.7)	

*P* value was estimated by chi‐square test or Fisher's exact test, in some cases the cell value <5%.

a Responses to self‐rated health status were dichotomized into two groups: excellent/very good/good (more than good) versus poor/very poor (not good).

b For attitude toward dying and death, a “strongly agree” or “agree” response to a question was considered positive.

In backward‐selected multivariable logistic regression analyses (Table 4), we included factors that were associated in univariate analysis with negative attitude toward EPC. We constructed two models: model 1 for patients and model 2 for caregivers. In model 1, negative attitude toward EPC was statistically significantly associated with male gender (adjusted odds ratio [aOR], 2.26; 95% confidence interval (CI), 1.58–3.24), age <50 years (aOR, 1.83; 95% CI, 1.26–2.64), early cancer stage (aOR, 1.61; 95% CI, 1.12–2.32), ICU admission (aOR, 1.51; 95% CI 1.00–2.28), and belief regarding preparing to practice charity (aOR, 2.47, 95% CI, 1.51–4.04). Model 2 showed that negative attitude toward EPC was significantly associated with not believing death is painful and should be feared (aOR, 1.60; 95% CI, 1.06–2.42) and not thinking people should be remembered (aOR, 1.85; 95% CI, 1.02–3.39). Lower educational level showed an inverse association with negative attitude toward EPC in caregivers.

**Table 4:** Factors associated with negative attitude toward early palliative care by sociodemographic and clinical factors, self‐rated health status, and attitudes toward dying and death

Factor	Variables	Negative attitude toward early palliative care (ref: positive attitude)					
		Patient (Model 1)			Caregiver (Model 2)		
		aOR	95% CI	*P* value	aOR	95% CI	*P* value
Sex	Female	1 (Ref)					
	Male	2.26	1.58–3.24	<0.001			
Age	≥50	1 (Ref)					
	<50	1.83	1.26–2.64	0.001			
Education	Middle school or less				1 (Ref)		
	High school				0.22	0.18–0.41	<0.001
	College or higher				0.29	0.16–0.53	<0.001
Stage	Advanced	1 (Ref)					
	Early	1.61	1.12–2.32	0.011			
Experience of ICU admission	No	1 (Ref)					
	Yes	1.51	1.00–2.28	0.048			
Attitude toward dying and death							
Death is painful and therefore to be feared	Positive				1 (Ref)		
	Negative				1.60	1.06–2.42	0.024
Dying people should prepare to practice charity	Positive	1 (Ref)					
	Negative	2.47	1.51–4.04	<0.001			
People should be remembered	Positive				1 (Ref)		
	Negative				1.85	1.02–3.39	0.044

aOR, adjusted odds ratio; CI, confidence interval; EPC, early palliative care; ICU, intensive care unit; Ref, reference.

Backward‐selected multivariable logistic regression analysis, with sl stay = 0.05, including variables identified as independent predictors that showed statistical significance of *P *<* *0.10 in univariate analysis.

Model 1 included variables significant in univariate analysis: sex (female vs. male), age (≥50 vs. <50), job (yes vs. no), religion (yes vs. no), stage (advanced vs. early), experience of ICU admission (yes vs. no), self‐rated physical, mental, and general health status (more than good vs. not good), and attitude toward death as preparing to practice charity (positive vs. negative).

Model 2 included variables significant in univariate analysis: age (≥50 vs. <50), level of education (middle vs. high vs. college or more), monthly income level (in 1000 Korean won) (<2000 vs. 2000–4000 vs. >4000), self‐rated mental, social, and spiritual health (more than good vs. not good), and attitude toward death as the ending of life, fearful one, being remembered (positive vs. negative).

## Discussion

Our study showed that a significant portion of both patients with advanced cancer and family caregivers showed positive attitudes toward EPC. A high proportion of positive responses indicate that these groups are eager to receive EPC, even if EPC is not introduced as a routine clinical practice in Korea yet. However, more than 10% of participants did not regard EPC as a necessary component for cancer management, and the reasons for negative attitudes toward EPC differed between patients with cancer and caregivers. These findings suggest that further implementation could be designed to target those barriers specifically.

Despite the improvements in quality of life, psychological distresses, symptom burden, and prognostic understanding provided by EPC, there are many barriers against EPC in patients with advanced cancer. Among them, patient and caregiver perception of EPC is important because it can prevent or alter use of the service. Nevertheless, most studies on attitudes and barriers to EPC have focused on healthcare providers rather than adult patients and caregivers 11, 17, 18. Studies on attitudes toward PC among patients with cancer and families have been widely performed 19, but that literature is mostly limited to PC during the end stages for patients with terminal cancer. Attitudes toward EPC should be distinguished from those toward end‐of‐life PC because EPC is not just a simple combination of PC and anticancer treatment. For patients and families, these results mark a transition from the belief that PC is provided only when cancer treatment is over to a new paradigm in which EPC is considered a part of the early treatment process in a comprehensively integrated care plan 5.

The high proportion of positive responses toward EPC in our study is consistent with the results from a study on perceptions of EPC in pediatric patients with cancer and their families 13. That study demonstrated that very few children or parents expressed oppositions to EPC or perceived barriers to EPC, and patients showed more positive expectations for symptom control and the integrative role of EPC than their parents. Those results differ from the expectation that patients and caregivers fear that they will lose hope or hesitate to continue treating their disease if they receive EPC.

However, a fair number of patients and caregivers answered that they did not want EPC because EPC may be an obstacle to cancer treatment or because PC is not beneficial. A serious misconception that PC is only end‐of‐life care and hinders anticancer treatment inculcates a negative impression in patients and family and influences them to refuse early integration of PC until treatment is finished 4, 20. To successfully change this thought, we suggest two possible solutions. First, there is a need for physician education to improve their understanding of the goals and significance of EPC 18, 20. Many studies found that health professionals lack knowledge of EPC and are afraid that their patients think oncologists will give up on them in the early stage or that they will miss a chance to participate in clinical trials if they participate in EPC 18. In a European study 11, only 22% of lung cancer specialists reported that they would refer patients to specialized PC at an early stage. Second, primary referring physicians should effectively communicate the goals of EPC to patients and family at the time of referral and actively engage them in EPC. Palliative care communication can be strenuous for physicians. According to the *Patient‐Clinician Communication Consensus Guideline* from the American Society of Clinical Oncology 21, successful communication skills to help patients make decisions should include the following: (1) Clinicians should explore the patient's goals, needs, and priorities; (2) clinicians should provide information about the risks and benefits of all possible options and explain the goal of care; (3) clinicians should check for patient and family concerns; and (4) clinicians should respond empathically to verbal and nonverbal patient actions. To reduce patient anxiety and reluctance before decision‐making, we also recommend that clinicians provide assurance that anticancer care will continue. Role‐playing simulation might be helpful to provide training for these skills.

Another major issue causing negative attitudes toward EPC is the additional financial burden that patients or caregivers might suffer. Interestingly, the proportion of respondents who gave financial burden as a reason for negative attitude was higher in caregivers than in patients. This reflects the high rates of financial burden on Korean family caregivers, who are usually responsible for the cost of treatment 22. The National Health Insurance system in Korea provides universal coverage for all citizens. Because PC was first based on the “Cancer Control Act” enacted in 2003, it has only been reimbursed when provided as end‐of‐life hospice care 23, 24, which is covered by National Insurance. In Korea, PC was initially provided as an isolated treatment practice or according to the congress practice models presented by Bruera and Hui 3, but the integrated care model has recently increased in popularity. For example, a total of 20 hospitals will offer PC team service in 2018 25, and this service is expected to continue to increase. Nevertheless, PC at earlier stages is not currently covered by insurance, and a patient or caregiver is required to pay for the service themselves. However, some studies revealed that EPC is more cost‐effective than standard oncology care alone 26, 27 and decreases the overall financial burden by eliminating unnecessary and futile inpatient costs 7, 27, 28. Therefore, a systematic approach to implement financial support should precede the institution of EPC.

Factors related to the negative attitudes toward EPC varied between the groups. This suggests that the perceived potential benefits of EPC might be different between patients and caregivers. Our findings showed that male and younger patients showed more negative attitudes for EPC than female and older patients. This may reflect the known tendency that male 29 and younger 30 patients prefer aggressive care to PC. However, according to a study of the efficacy of EPC intervention 31, these groups appeared to experience more improvement of quality of life and depression with EPC than other patients did, suggesting that they should be involved in EPC. Interestingly, patients who experienced ICU admission showed more negative attitudes toward EPC than those who did not. We speculate that after ICU care, survivors might consider aggressive care as more beneficial than PC. The correlations between attitudes toward death and attitudes toward EPC reported in our study imply that beliefs about death and dying may differently influence the perception of EPC. Groups that did not agree with EPC also showed negative thoughts on charity upon death or being remembered. Patients not afraid of death were also less likely to be interested in EPC. Finally, the caregiver's educational status appears to affect EPC in a negative way. Educational status is an important factor related to attitudes toward aggressive care such as advanced directives 32 and other components of PC 33, 34. Considering socioeconomic status would be crucial to identify attitudes toward EPC.

This study has several limitations. First, it was conducted in single nation, so cross‐cultural differences should be considered to interpret the findings. Second, this is a cross‐sectional study, which makes it hard to determine a cause–effect relationship. Further prospective study is needed to explore attitudes toward EPC. Third, we did not include the specific types of anticancer treatment that patients received, which might influence the preference of EPC. Fourth, at the time of the study initiation, EPC was not included in routine oncology practice. After EPC is widely introduced, attitudes toward EPC might be different. Finally, there is a possibility that the participants had a different understanding of the concept of PC because we did not confirm their own definition of PC in the questionnaire. We have attempted to provide a clear and exact meaning of PC for participants to accept by defining PC in the questionnaire, but this may have influenced the attitudes of the participants toward EPC.

In conclusion, the concept of EPC can generally be accepted by adult patients with cancer and family caregivers. However, there are still a non‐negligible proportion of people who has negative attitudes toward EPC. Physicians’ communication with patients and caregivers and financial support would help them acknowledge the importance of EPC.

## Conflict of Interest

The authors indicated no potential conflict of interests.
